# Effectiveness of targeting fathers for breastfeeding promotion: systematic review and meta-analysis

**DOI:** 10.1186/s12889-018-6037-x

**Published:** 2018-09-24

**Authors:** Pasyodun Koralage Buddhika Mahesh, Moraendage Wasantha Gunathunga, Suriyakumara Mahendra Arnold, Chintha Jayasinghe, Sisira Pathirana, Mohamed Fahmy Makarim, Pradeep Malaka Manawadu, Sameera Jayan Senanayake

**Affiliations:** 1 Office of Regional Director of Health Services, Colombo, Sri Lanka; 2 Department of Community Medicine, Faculty of Medicine, University of Colombo, Colombo, Sri Lanka; 3 Epidemiology Unit, Colombo, Sri Lanka; 4 Faculty of Medicine, University of Colombo, Colombo, Sri Lanka; 5 Ministry of Health, Colombo, Sri Lanka; 6 Post Graduate Institute of Medicine, Colombo, Sri Lanka

**Keywords:** Breastfeeding, Fathers’ influence on breastfeeding, Exclusive breastfeeding, Breastfeeding promotion, Infant nutrition, Partner support in breastfeeding

## Abstract

**Background:**

Further research gaps exist in relation to the promotion of breastfeeding. Robust scientific evidence obtained by a meta-analysis would provide objectively summarized data while enabling the assessment of consistency of findings. This review includes the first documented meta-analysis done on the effectiveness of targeting fathers for promoting breastfeeding (BF). Assessments have been done for a primary outcome and for six more secondary outcomes.

**Methods:**

PubMed, EMBASE, Google Scholar, CENTRAL databases and unpublished researches were searched. Selections of randomized-controlled trials and quasi-experimental studies were done in three rounds. Heterogeneity and potential publication bias were assessed. Eight studies were included in meta-analysis and others in narrative synthesis of the outcomes. Pooling was done with the Mental- Haenszel method using risk ratio (RR). Summary-of-Findings table was composed by Review-Manager (version 5.3) and GRADEproGDT applications. Subsequent sensitivity analysis was done.

**Results:**

Selected eight interventional studies included 1852 families. Exclusive BF at six months was significantly higher (RR = 2.04, CI = 1.58–2.65) in the intervention groups. The RR at 4 months was 1.52 (CI = 1.14 to 2.03). Risk of full-formula-feeding (RR = 0.69, CI = 0.52–0.93) and the occurrence of lactation-related problems were lower in the intervention groups (RR = 0.24, CI = 0.10–0.57). More likelihood of rendering support in BF-related issues was seen in intervention groups (RR = 1.43, CI = 1.22–1.68). Increase of maternal knowledge and favorable attitudes on BF were higher in the intervention groups (*P* ≤; 0.001). The quality of evidence according to GRADE was “low” (for one outcome), “moderate” (for four outcomes), and “high” (for two outcomes).

**Conclusions:**

Targeting fathers in promotion of BF has provided favorable results for all seven outcomes with satisfactory quality of evidence.

This review was registered in the PROSPERO-registry (ID: 2017-CRD42017076163) prior to its commencement.

## Background

Breast milk is regarded as the best source of nutrition a newborn can get [1]. It provides favorable outcomes to the baby as well as to the mother [2–6]. These outcomes are not only limited to growth-related, immunological, and economic benefits, but also extend to a larger scope with the assumption of influencing genetic-dynamics as well [7]. Furthermore, breastfeeding has been found to be associated with favorable adult outcomes like prevention of chronic non-communicable diseases which are becoming global epidemics [8–11]. The World Health Organization (WHO) and the United Nations Children’s Fund (UNICEF) recommend an exclusive breastfeeding (EBF) period of 6 months for all settings [1, 12–14]. Since 2001, this recommended duration has not been changed until now [15, 16]. Yet in many communities, the breastfeeding norms are being challenged [17, 18]. It has been mentioned that in certain settings of low and middle-income countries, the cumulative prevalence of EBF in babies younger than 6 months is less than 40% [6]. In many of high-income countries, the duration of EBF period is shorter compared to resource-poor settings [6].

There are certain factors that increase as well as decrease the duration of EBF. Lack of family and social support have been determined as detrimental factors associated with the exclusivity and duration of breastfeeding [1, 19]. In addition, the occurrence of lactation-related problems like breast engorgement, sore nipples, incorrect attachment, and promotion of formula feeding have been recognized as negatively influencing factors of breastfeeding [20, 21]. In contrast, higher knowledge of mothers on breastfeeding helps to promote it [20].

Promotion of breastfeeding needs multilevel supportive measures with interventions being implemented through several channels [17]. Fathers have been named as one recommended target in promoting breastfeeding [22, 23]. Qualitative research findings have revealed some domains of the father’s role in breastfeeding [24]. Yet, literature suggests that “fathers or male partners” have not been given adequate emphasis in the promotion of breastfeeding [18, 25].

The available research findings of observational studies point towards a positive correlation between the support of the male partner and the likelihood of continuation of breastfeeding [18, 25, 26]. Even the perceived support of the partner is linked with favorable levels of self-efficacy [27]. In addition to their support, the attitudes of the husband have been documented as determinants of breast feeding self-efficacy [28, 29]. In contrast, some literature does not recommend the “broad application of male involvement” in promoting EBF [30]. A cohort study has documented that though their emotional support does, the practical support of fathers as not being associated with better breastfeeding [31]. Similarly, a study done in India has revealed that though the fathers’ attitudes support breast feeding, they do not influence the duration of EBF [32].

Child nutrition programs require much more investments and commitments globally [13]. At the same time, more scientific literature is needed in determining the effectiveness of interventions on breastfeeding as there are issues on the generalizability of currently available evidence [33]. The WHO has highlighted that more scientific evidences are needed “across different regions, countries, population groups and contexts, in order to adequately and sensitively protect, promote and support breastfeeding.” [34]. Meta-analysis of systematically reviewed data would provide objectively-summarized precise data and enable assessing the consistency of findings [35]. It is recommended that the use of randomized control studies as an ideal strategy in determining the effectiveness of interventions which target breastfeeding [36, 37]. When randomization is not possible, quasi-experimental studies may produce better evidence than observational studies in evaluating the effectiveness of interventions [38, 39].

The present review included assessing a primary outcome as well as six secondary outcomes. First, a specific objective was to conduct a meta-analysis on its effectiveness on the adherence to EBF practices at the end of 6 months as the primary outcome. The second specific objective included six other secondary outcomes which are complementary parameters in determining the effectiveness of breastfeeding or factors which significantly influence the primary outcome. They were: EBF at the end of 4 months, full formula-feeding within 2 months, support of the father, prevalence of breastfeeding related problems, knowledge of the mother on breastfeeding, and the attitudes of the mother on breastfeeding.

## Methods

### Protocol and registration

Preferred Reporting Items for Systematic Reviews and Meta-analyses (PRISMA) guidelines were referred [40]. The review was registered in the PROSPERO-International prospective register of systematic reviews registration (2017-CRD42017076163). Subsequent amendments were made in the protocol clarifying the eligibility criteria further.

### Eligibility criteria

The research question was composed based on PICOS and SPIDER sequence [41]. It was formulated as “targeting the father/male-partner in addition, more effective than targeting the pregnant or new mother alone, in promoting breastfeeding with evidences of interventional studies.”

The criteria for the selection of studies included: being a randomized or quasi experimental study, the intervention group including the male partners, and the intervention being delivered either in the antenatal period and/or within the postnatal period.

### Search strategy

The PubMed, EMBASE, Google Scholar and CENTRAL-Cochrane library were searched. Our search strategy was ‘breastfeeding’ AND ‘expectant father OR father OR male partner.’ By contacting the fellow colleagues of the related fields, attempts were made to seek any unpublished literature. Furthermore reference lists of the selected articles and references of the systematic reviews were carefully studied in tracing the eligible articles.

### Selection of studies

The selection of the studies was done in three rounds. In the first round, original research articles, which were compatible with the general objective of the present review, were selected. In the second round, articles on experimental studies were retained. In the third round, the studies which are compatible with the specific objectives were retained. In the first and second rounds, when the selection could not be done by the details mentioned in the abstracts, full articles were referred. Full articles were compulsorily referred in the third round.

**Fig. 1:**
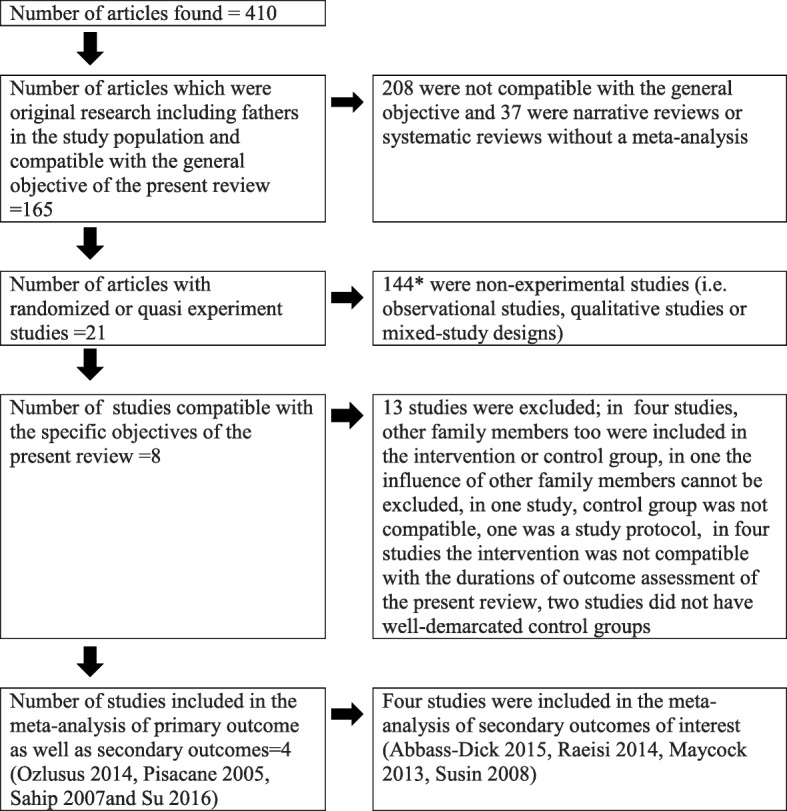
Flow-diagram on the selection of studies

The selection of articles was done by two independent reviewers. It was ensured that in selected articles, the intervention had been specifically targeting breastfeeding promotion. The articles which were selected by both reviewers were identified first. When there was a disparity, a third reviewer was involved to resolve it following a discussion with original reviewers. Following de-duplication, 410 articles were selected to be screened (Fig. 1).

### Data-extraction

Data extraction was done by two reviewers independently using a pre-designed template. A third reviewer ensured the similarity of the two datasets of the initial reviewers. The extracted variables are summarized in Table 1.

**Table 1:** Extracted variables from selected studies

Population	Intervention	Comparison	Outcome	Study design
-Eligibility criteria -Characteristics of the participants	-Kind of intervention/s done with duration/s	-Number of arms -Number participated -Number completed the allocated exposure	-Primary and secondary outcomes -How the outcomes were measured -Number of participants with each outcome	-Type of design -Year of conduct -Study setting

### Estimation of bias

The risk of bias table was composed based on the recommendations for the randomized trials and quasi experimental studies [42–47]. The bias assessment was based on the methodological issues related to random number generation, allocation concealment, performance bias, detection bias, attrition bias, reporting bias, and any other bias. Determination of the level of bias was done by two reviewers independently and was contributed by a third reviewer in case of a disparity of decisions. Publication bias was assessed by a funnel plot [48].

### Meta-analysis and narrative synthesis

The assessment for statistical heterogeneity of the selected studies was done with chi-square and I-square tests for all the meta-analyses [48, 49]. The cut-off of the I-square test for heterogeneity was considered as 50% and for the *p* value of chi-square test was considered as 0.1 [42, 48]. Meta-analysis was done by the software Review Manager (version 5.3) having done the heterogeneity assessments [50]. Mental- Haenszel method was used in pooling. Fixed-model assumptions were used in meta-analysis which was complemented by the random-model assumptions in assessing the robustness of findings. Risk Ratio (RR) was used as the effect measure as it has been described as less-misleading compared to the Odds Ratio [51, 52]. Narrative synthesis was done when the selected studies were found be heterogeneous by both chi-square statistic and I-square values [53, 54]. Combined results were presented in a Summary of Findings (SoF) table (Table 2) [55]. The quality of evidence was assessed with the criteria of “Grading of Recommendations, Assessment, Development and Evaluations (GRADE) Working Group” with the help of the “GRADEproGDT” application [45–47].

**Table 2:** Summary of Findings (SoF) Table

Effectiveness of targeting fathers for breast feeding promotion						
P - Expectant and new fathers I – Health education on breast feeding C – Educating only the mother O – Promotion of breast feeding						
Outcomes	Assumed risk^a^	Corresponding risk^b^	Relative effect	No. of participants (studies)	Quality of evidence GRADE	Comments
Exclusive BF for six months	201 per 1000	411 per 1000 (318 to 534)	RR = 2.04 (1.58–2.65)	587 [71–74] (4 studies)	Moderate^c^	Meta-analysis done
EBF for four months	194 per 1000	294 per 1000 (221 to 393)	RR = 1.52 (1.14 to 2.03)	507 [72, 73, 77] (3 studies)	Low^d^	Meta-analysis done
Full formula feeding within two months	247 per1000	170 per 1000 (128 to 230)	RR = 0.69 (0.52 to 0.93)	721 [73, 76] (2 studies)	Moderate^e^	Meta-analysis done
Support of the father	548 per 1000	783 per 1000 (668 to 920)	RR = 1.43^f^ (1.22 to 1.68)	383 [73, 75, 78] (3 studies)	High^g^	Meta-analysis done for two studies [75, 78] and narrative synthesis done for one study [73]
	In the third study (Su 2016), fathers in the intervention group knew how to support continuation of breast feeding. When a breastfeeding related problem occurred, they provided solid support. The fathers in the control group did not know how to support even if they wanted to.					
Knowledge of the mothers on breast feeding (BKS scale and a developed tool)	In one study (Su 2016), mothers’ knowledge on breastfeeding increased by 19.75 points in the experimental group and by 14.81 in the control group (*p* = 0.009). In another study (Raesi 2014), the pre-study knowledge was not significantly different in the two groups whereas the post-intervention knowledge was 103 in the experimental group and 95.71 in the control group (*P* < 0.0001).			169 [73, 75] (2 studies)	Moderate^h^	Narrative synthesis done
Breast feeding related problems	179 per 1000	43 per 1000 (18 to 102)	RR = 0.24 (0.10 to 0.57)	280 [71] (1 study)	High^i^	Narrative synthesis done
Maternal attitudes towards breast feeding (IIFAS scale)	The increase of maternal attitudes towards breastfeeding was significantly more in the intervention group (*p* = 0.001)			69 [73] (1 study)	Moderate^j^	Narrative synthesis done

^a^Total events divided by the total participants in the control group

^b^Function of “assumed risk” and the “relative effect”

^c^Quality was downgraded due to risk of bias (2 points) and upgraded for large effect (1 point)

^d^Quality was downgraded due to risk of bias (2 points)

^e^Quality was downgraded due to risk of bias (1 points)

^f^Meta-analysis was done for findings of two studies as the third was heterogeneous

^g^Quality was downgraded due to risk of bias (1 points) and upgraded for large effect (1 point)

^h^Quality was downgraded due to risk of bias (2 points) and upgraded for large effect (1 point)

^i^Quality was downgraded due to risk of bias (1 points) and upgraded for large effect (1 point)

^j^Quality was downgraded due to risk of bias (2 points) and upgraded for large effect (1 point)

### Assessing the robustness of the results

The sensitivity analysis was done by re-performing the meta-analysis with random-model assumption following the initial fixed-model assumption [56, 57]. Furthermore, it was done by repeating the meta-analysis of the primary outcome leaving out one study at a time [58].

## Results

### Selection of studies

The selection-related details of the studies are summarized in Fig. 1. Twenty experimental studies were thus selected in the second round. Out of these twenty, thirteen studies were excluded in the third round [20, 59–70]. Out of the remaining eight studies, four were included in the meta-analysis of the primary as well as secondary outcomes of interest [71–74]. Four others were included in meta-analysis of the secondary outcomes [75–78]. All eight studies included 1852 families. During the search, only one article which gives the impression of an experimental study by the title, could not be traced [79].

### Interventions of the studies

Interventions consisted of Information-Education-Communication methodologies including; face-to-face discussions, power-point presentations, usage of brochures, usage of models, leaflets, and electronic media. In three studies (Su 2016, Sahip 2007, Raeisi 2014) the intervention was done during the antenatal period [73–75]. In two studies (Ozlusus 2014, Maycock 2013) the intervention started in the antenatal period and extended in to the neonatal period [72, 76]. In three studies (Susin 2008, Pisacane 2005, Abbass-Dick 2015), the intervention was done in the neonatal period [71, 77, 78].

### Results of bias assessment

**Fig. 2:**
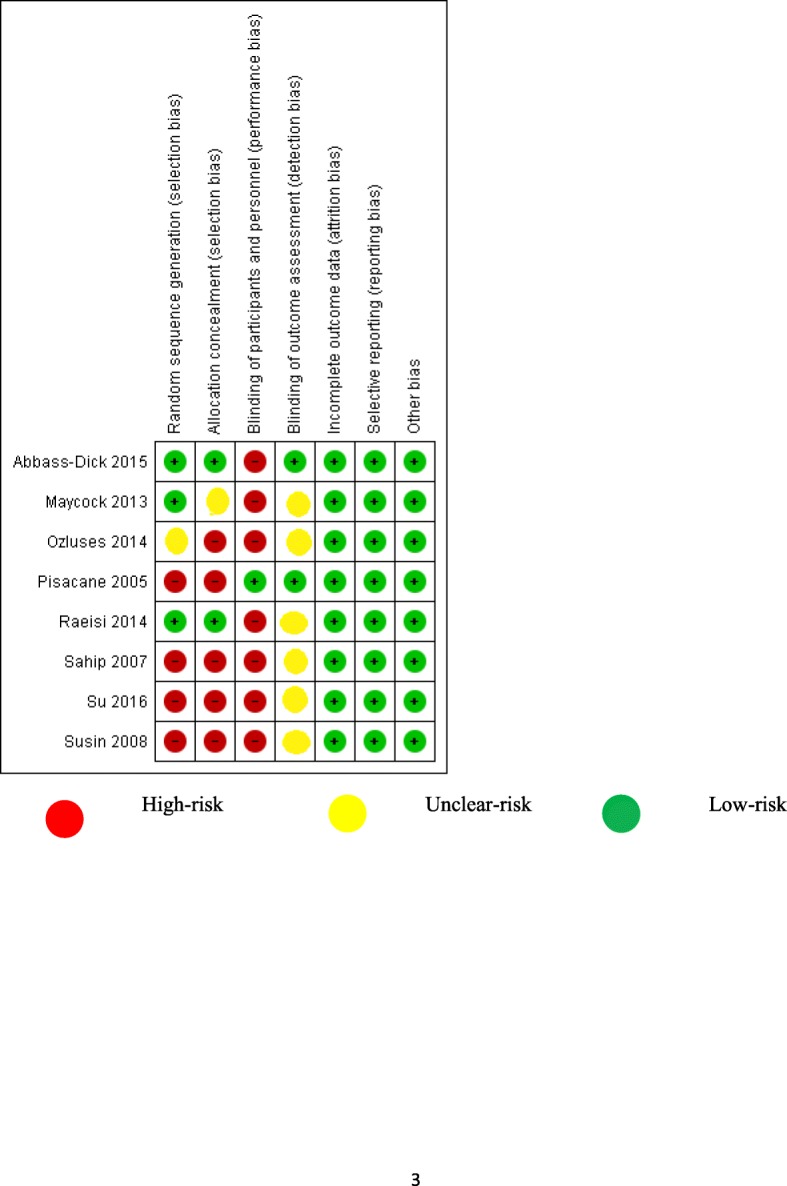
Risk of bias summary of individual studies

All eight studies were with low risks of attrition, reporting, and other biases. Blinding of participants was done only in the study of Piscane (2015). High-risk of bias due to issues related to random number generation and allocation concealment could not be excluded respectively in four and five studies (Fig. 2).

### Meta-analysis of the primary outcome

**Fig. 3:**
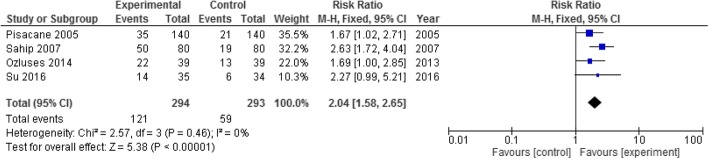
Forest-plot with fixed-model assumption for the primary outcome

Four studies were selected for the meta-analysis for the first specific objective (i.e. primary outcome) (Table 3). This meta-analysis included 587 families with 294 in the experimental group (i.e. the male partner involved in the health education) and 293 in the control group (male partner not involved in the health education). The four selected studies seemingly were not significantly heterogeneous (I^2^ = 0%, *p* value = 0.46). The pooled RR was 2.04 (CI = 1.58 to 2.65). It reflects that compared to a baby whose father has not participated in a breastfeeding promotional intervention, a baby whose father attended such an intervention is more than two times as likely to be exclusively breastfed for 6 months (Fig. 3).

**Table 3:** Characteristics of the selected studies for the meta-analysis of the primary outcome

Study	Study	Population	Comparison	Intervention	Outcomes
1.Ozlusus, 2014 [72]	Turkey, Experimental study	-Ability to read, write and speak Turkish -Who were hoping to live in a mentioned area in Turkey until infants would be six months	1. Control group (No intervention) with 39 families 2. Experimental group I (Only mothers) with 39 participants 3. Experimental group II (Both mom and dad) with 39 families	Education manuals, demonstrations. Fathers’ education done during visiting hours (from the day mother got admitted until the day of discharge)	-Exclusive breast feeding: Control- 12.8%, Group I- 33.3%, Group II- 56.4
2. Pisacane, 2005 [71]	Italy, A controlled trial	Parent-pairs of healthy, term normal birth weight infants. unmarried women,	1. Intervention group - 280 participants (140 mothers and 140 fathers) 2. Control group B- 280 participants (140 mothers and 140 fathers)	Intervention included a face-to-face 40 minute session on infant feeding, difficulties in breast feeding including their management. A leaflet given at the end.	-Full Breast feeding at six months: Intervention group-25%, Control- 15%
3. Sahip, 2007 [74]	Turkey Interventional study.	Expectant fathers attending the workplaces in which a trained physician was available consisted the intervention group.	1.Intervention, 80 2. Control, 80	Six sessions each of 3-4 hours. A certificate to hang on newborn babies’ room.	-Full Breast feeding at six months: Intervention group-62.5%, Control- 23.7%
4. Su, 2016 [73]	China, Quasi-experimental study	Participants fluent in Mandarin, more than 20 years, first pregnancy, singleton fetus, couple living together, gestational age more than 39 weeks	-Intervention group with 36 pregnant mother-husband pairs -Control group of 36 with only the pregnant mother	60-90 minute health education sessions using power-point presentations and models	-Full Breast feeding at six months: Intervention group- 14 of 35 (40%), control group-6 of 34(17.6%)

**Fig. 4:**
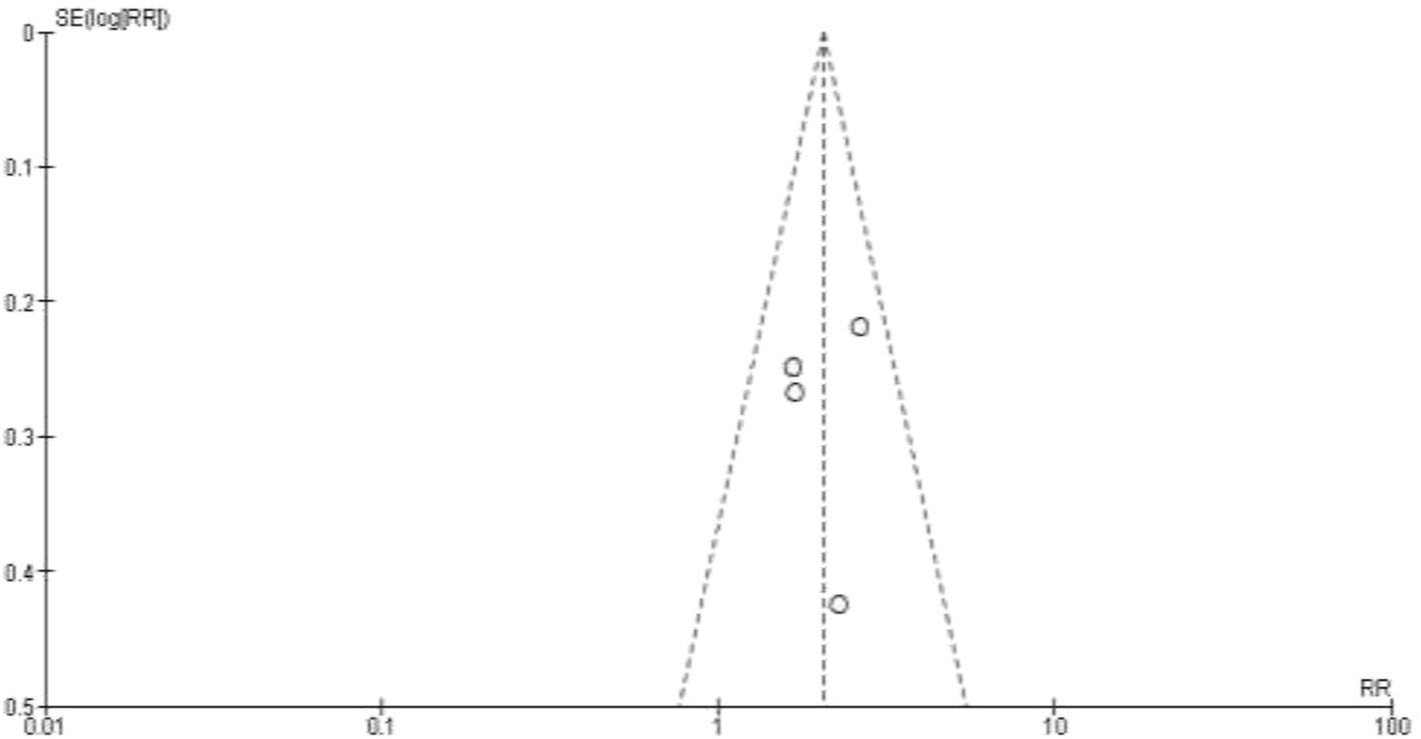
Funnel plot of the studies used in meta-analysis of the primary outcome

The effect measures of the selected studies yielded an approximately symmetrical Funnel-plot as shown in Fig. 4.

The results of the sensitivity analysis have been mentioned in Table 4. Even when the meta-analysis was done with random-model assumption as well as when it was repeated removing one study at a time, the pooled estimates were significantly favoring the interventional groups.

**Table 4:** Sensitivity and sub group analysis of the pooled estimate of primary outcome

	Heterogeneity	Number	Risk Ratio	Confidence Interval
With fixed-model assumption (four studies)	I^2^ value- 0% *P* = 0.46	587	2.04	1.58 to 2.65
With random-model assumption (four studies)	I^2^ value- 0% *P* = 0.46	587	2.04	1.57 to 2.65
Fixed-model assumption with Ozlusus 2014 removed	I^2^ value- 0% *P* = 0.38	509	2.14	1.59 to 2.89
Random-model assumption with Ozlusus 2014 removed	I^2^ value- 0% *P* = 0.38	509	2.17	1.61 to 2.93
Fixed-model assumption with Pisacane 2005 removed	I^2^ value- 0% *P* = 0.44	307	2.25	1.66 to 3.07
Random-model assumption with Pisacane 2005 removed	I^2^ value- 0% *P* = 0.44	307	2.21	1.63 to 3.01
Fixed-model assumption with Sahip 2007 removed	I^2^ value- 0% *P* = 0.81	427	1.77	1.27 to 2.46
Random-model assumption with Sahip 2007 removed	I^2^ value- 20% *P* = 0.29	427	1.76	1.27 to 2.44
Fixed-model assumption with Su 2016 removed	I^2^ value- 0% *P* = 0.29	518	2.02	1.53 to 2.66
Random-model assumption with Su 2016 removed	I^2^ value- 0% *P* = 0.29	518	2.00	1.47 to 2.73

### Meta-analysis and narrative synthesis of the secondary outcomes

The characteristics of the selected studies for the analysis of secondary outcomes have been summarized in Table 5. The SOF table was prepared for the seven outcomes (Table 2). For four outcomes (EBF at the end of 6 months, EBF at the end of 4 months, full formula feeding within 2 months and support of the father), meta-analysis showed favorable outcomes in the interventional groups than in the control groups. Respectively, the chi-square values and I-square values in the heterogeneity analysis for EBF at the end of 4 months were 0.62 and 0%. For formula feeding within 2 months the respective values were 0.13 and 55%. For the support of father, they were 0.37 and 0%, respectively.

**Table 5:** Characteristics of the selected studies for the meta-analysis of the secondary outcomes

Study	Study	Population	Comparison	Intervention	Selected outcomes
1. Abbass-Dick 2015 [78]	Canada, Randomized controlled trial	Primiparous mothers with a singleton birth, 18 or more years old, term delivery, with a male partner	1. Intervention group (107 couples) 2. Control group (107 couples)	Face to face intervention of approximately 15 minutes. Could refer to materials like workbook, video and a website later	-Support of fathers: Intervention group- 71%, Control group- 52%
2. Raeisi 2014 [75]	Iran, Interventional study	Mother in second trimester, no pregnancy complication or underlying disease	1. Intervention group (Group A) – 50 couples 2. Control group (Group B)- 50 couples	Three training sessions including brochures	-Support of fathers: Intervention group-94%, Control- 60% -Knowledge of mothers Intervention group-103 points, Control- 95.71 points
3. Maycock 2013 [76]	Australia Randomized controlled trial	Mother must be more than 18 years, father must be contactable living in Western Australia and willing to involve in child rearing	1. Intervention group- 358 2. Control group-298	Two hour antenatal health education session and a postnatal social support package including printed materials.	-Full formula feeding at six weeks: Intervention group-18.4%, Control- 24.8%
4. Susin 2008 [77]	Brazil, Controlled clinical trial	Couples living in the city of Porto Alegre, infants have no health problems, birth weight equal to or more than 2500 g, have initiated breastfeeding	-Control group (no intervention)- 201 -Intervention group with mother- 192 -Intervention group with both mother and father- 193	Health education session with a 18-minute video and a discussion, an explanatory handout was provided	-EBF at four months: Intervention group with mothers- 11.1%, Intervention group with both mother and father-16.5%

The first three-pooled measures were found to be robust with sensitivity analysis done by repeating with random-model assumption. The effect measure for the “support of the father” became non-significant when the latter model-assumption was used. The occurrence of breastfeeding problems showed a very lower likelihood in the intervention group. The narrative summaries for knowledge of mother on breastfeeding, maternal attitudes towards breastfeeding and the additional narrative summary for the support of father, too demonstrated favorable outcomes in the intervention groups than in the control groups.

The quality of evidence was determined as: low (for one outcome), moderate (for two outcomes), and high (for two outcomes) based on the recommendations of GRADE recommendations.

## Discussion

This is the first systematic review with a meta-analysis of the effectiveness of targeting the male partner for the promotion of breastfeeding. The meta-analysis revealed that targeting the fathers is associated with two times the likelihood of getting the baby exclusively breastfed for 6 months. Furthermore, the experimental groups achieved favorable results when the other six outcomes were concerned as well. The systematic review included both randomized controlled trials as well as quasi-experimental trials. The suitability of the inclusion of the latter in systematic reviews have been highlighted in modern literature [80].

The quality of this systematic review and meta-analysis was ensured with several steps. Firstly, the data search was done without restricting to a time period. It is mentioned that searching all available data as a better strategy in systematic reviews [81]. The duration of EBF was recommended to be 6 months from the beginning of twenty-first century and until then, there had been a debate about shorter duration [82, 83]. Many of the previously documented literature had no uniformity of considering EBF period as 6 months [84]. Since then it was decided to include another secondary outcome for a shorter duration (i.e. EBF for 4 months).

Risk of bias estimates were not done by averaging the several components of the bias estimates but by utilizing recommended guidelines. [42, 45, 85] Not surprisingly, in the majority of studies, blinding had not been possible and as a result they were categorized as high-risk for the performance bias. Strict categorizing criteria were adhered to in the estimation of bias. As an example, when the allocations were done based on time period of delivery of baby, the high-risk categorization for selection bias was given and down-grading was done in determining the quality of evidence in the SoF table. To improve the validity of the results, RR was used as the effect measure instead of odds ratio [51, 52].

It is recommended to consider the statistical, clinical, and methodological heterogeneity in interpreting the quality of meta-analysis [48, 86, 87]. The statistical heterogeneity of the studies were seemingly acceptable based on I-square percentages and chi-square values [42, 48–50]. Only the I-square value was marginally high for one of the secondary outcomes (i.e. for the support of father). Clinical heterogeneity does not seemingly influence the results as most of the outcomes for which the meta-analyses were done are objectively categorically coded (as an example, whether EBF continued for 6 months or not). In all studies, the periods of interventions did not extend beyond the neonatal period. All outcome measurements had been done after 2 months of the birth. Measures to minimize methodological-bias were adhered to in selection of the studies and in the expression of risk of bias of each study. Furthermore, the control groups of the studies had been recruited from similar settings. Even though “fatherhood” is influenced by the culture, universally, fathers care for the well-being of the family and children [88, 89]. Hence, though the degree of involvement of a father may vary in nutrition related affairs of the newborn, its direction in all settings can be assumed as towards getting more benefits to the baby.

An extensive sensitivity analysis has been done in the present manuscript. Sensitivity analysis has been defined as “a method to determine the robustness of an assessment by examining the extent to which results are affected by changes in methods, models, values of unmeasured variables, or assumptions.” [90] Since all the measures except one in the sensitivity analysis point towards significant favorable effects of targeting fathers, the robustness of the conclusions become high.

Targeting fathers was effective in increasing likelihood of EBF at the end of 6 months as well as at the end of 4 months. RR for the EBF at 6 months is higher than that of the figure at 4 months and double as compared to the control group. In other words, it is associated with a higher probability of uninterrupted provision of breast milk enabling the child to get its benefits [1–6]. This finding can be evaluated further using the fourth outcome in the SoF table which is the support extended by the father for breastfeeding. High quality evidence was seen in the meta-analysis as well as the narrative synthesis showing the favorable influence of targeting fathers on the prospective support they render. When fathers get to know the scientific evidence on the benefits of breastfeeding, it can be postulated that they would encourage the partner to continue this course. This would have resulted in increasing their support as well as indirectly prolonging the duration of EBF.

The prevalence of full-formula-feeding was less in the intervention group. This finding can be discussed coupling to the sixth outcome (i.e. occurrence of breastfeeding related problems). When fathers are educated on breastfeeding, due to their support (i.e. fourth outcome), better positioning and attachment of the baby to the breast during feeding would be facilitated. Lesser lactation-related problems would ensure not opting for the formula milk. Furthermore, this would be facilitated by the fact that mothers’ knowledge and attitudes on breastfeeding becoming more favorable with the intervention (i.e. fifth and seventh outcomes).

The effect of the intervention on mothers’ knowledge and on favorable attitudes can be described with several explanations. The mutual discussions that occur in the household with the partners would improve mothers’ knowledge. Secondly, the positive perception of the partner’s attitude on breastfeeding would foster the mothers’ attitudes as well. This is compatible with the global literature [27].

In the present review, all seven outcomes, including EBF rates, were favorably influenced by targeting the expectant fathers for promotion of breast milk. This review adds value to the attempts made by the healthcare systems in involving expectant and new fathers in the interventions for the promotion of breastfeeding. More emphasis could be given for the male-partner domain of the awareness packages that are done in the ante-natal and neonatal periods for the promotion of breastfeeding. Since the breastfeeding is associated with mitigation of communicable diseases as well as prospective occurrence of NCDs, targeting fathers becomes a cost-effective strategy which yields the effects through prolonged duration of EBF. Breastfeeding related indicators are used to determine the improvement of the health status of a country [91]. Furthermore, longer duration of breastfeeding is recommended as a “smart investment” in achieving Sustainable Developmental Goals [92]. Hence the intervention of this review would ensure better placement of countries in relation to health indicators.

There were several limitations of the review. Firstly, the results could not be standardized for the quality of the interventions. This was because the review was done on different experimental studies which were done on different settings using different intervention packages. To compensate for its impact, seven outcome measures which are supposed to be linked with the awareness on general-aspects related to breastfeeding were selected. Furthermore in selecting the studies, special emphasis was given to the intervention-related details. Since the determination of the risk of bias and grading of the quality of evidence include judgmental decision-making, undue influence of being “subjective,” could not be totally excluded. Yet, several steps like independent assessment by several reviewers, contacting the GRADE support group for clarifications were done. Another is that, in measuring the mothers’ knowledge (fifth outcome), different tools were used. To minimize its impact, the narrative summary was made to focus on the “change of knowledge” rather than on the raw scores.

## Conclusions

Targeting fathers in the antenatal and postnatal periods of the baby: improves EBF at 6 months (RR = 2.04, CI = 1.58–2.65) and EBF at 4 months (RR = 1.52, CI = 1.14 to 2.03). In addition it decreases the probability of full-formula-feeding at 2 months (RR = 0.69, CI = 0.52 to 0.93) and the occurrence of breastfeeding related problems (RR = 0.24, CI = 0.10 to 0.57). Furthermore it increases the support extended by the father in breastfeeding related issues (RR = 1.43, CI = 1.22 to 1.68). Mothers’ knowledge on breastfeeding and the favorable attitudes on breastfeeding are augmented with the intervention done on fathers (*P* ≤; 0.001).

The conclusions are robust as suggested by the sensitivity analysis. The quality of evidence ranges from “low” to “high” for different outcomes.
